# The Lived Experience of Contemporary Trainee Nursing Associates: The Evolution of the New Role as a Pathway to Nursing. A Phenomenological Study

**DOI:** 10.1111/ijn.70030

**Published:** 2025-06-16

**Authors:** Shubhangi Sarwan, Kathryn Sethi, Victoria Skerrett, Samantha Peck, Katy Sutherland‐Hastings, Joanne Brooke

**Affiliations:** ^1^ School of Nursing and Midwifery, Faculty of Health, Education and Life Sciences, Ravensbury House Birmingham City University, City South Campus Birmingham UK; ^2^ School of Nursing, Midwifery and Health, Faculty of Health, Education and Life Sciences, Secole Building Birmingham City University, City South Campus Birmingham UK; ^3^ School of Nursing and Midwifery, College of Medical and Dental Sciences University of Birmingham, Edgbaston Birmingham UK; ^4^ College of Nursing and Midwifery, Faculty of Health, Education and Life Sciences, Ravensbury House Birmingham City University, City South Campus Birmingham UK

**Keywords:** focus groups, nursing associates, phenomenology, qualitative, trainee nursing associates

## Abstract

**Background:**

In 2016, a new role, the nursing associate, was implemented within the nursing workforce in England to support the shortfall of registered nurses and create a new pathway into nursing. This study aims to explore trainee nursing associates' lived experience to understand if the new role has been accepted and embedded in the nursing workforce.

**Design:**

This study used a qualitative inductive phenomenological design. The Consolidated Criteria for Reporting Qualitative Research checklist (COREQ) was adhered to in developing this paper.

**Methods:**

Semi‐structured focus groups were conducted with participants completing their nursing associate programme in one higher education institute. Four focus groups with 14 participants occurred between June and November 2021. Thematic analysis was completed as described by Braun and Clarke from an inductive phenomenological perspective.

**Results:**

Four themes were identified: (1) new opportunities and knowledge; (2) academic and practice support; (3) pressure within clinical placements; and (4) the need for continued education and training.

**Conclusions:**

The new role has supported widening participation in higher education institutions and an affordable professional nursing pathway. However, challenges, such as a lack of understanding of the nursing associate role, remain in both clinical practice and higher education institutes. The development of the nursing associate role across specialities has commenced, which inevitably causes further confusion.

What is known about this topic?
  - A new role, the nursing associate, was implemented in England in 2016 to support the shortfall of registered nurses.
  - Nursing associates are registered with the governing nursing body in England, the Nursing and Midwifery Council.
  - The implementation of nursing associates has been challenging due to a lack of acceptance and understanding by healthcare professionals and the emerging ambiguity of responsibilities across specialities.
What this paper adds?
  - The nursing associate role has widened participation in higher education and is an affordable professional nursing pathway.
  - Challenges remain for trainee nursing associations in both clinical practice and higher education institutions, which need to be addressed.
  - The development of the nursing associate role across specialities has commenced, which inevitably is causing confusion.
The implications of this paper:
  - The clinical identity of nursing associates needs to be further developed both across and within specialities.
  - The responsibilities of nursing associates need to be further developed both across and within specialities.

## 1 Introduction

The shortage of nurses worldwide is widely known (World Health Organisation 2020); in England alone, there are 43 339 registered nurse vacancies (NHS Digital et al. 2023). An approach to address the shortfall of nurses in England is the introduction of a new nursing role, the nursing associate (King et al. 2020). The status and duties of a nursing associates (NAs) are comparable to those of licenced practical nurses in Finland and North America and enrolled nurses in Australia, New Zealand, South Africa and other European nations (Robertson et al. 2023). Similar to these countries NAs are registered with the regulatory body of nursing in the United Kingdom, the Nursing and Midwifery Council (NMC). The only noticeable difference between nursing associates and nursing students during their education is nursing associates are referred to as learners and nursing students are referred to as students (Robertson et al. 2022).

The 2‐year full‐time NA apprentice programme includes an allocation of 20% of time for academic studies and 20% for clinical practice placements (external to current work‐base), whilst the remaining hours are allocated for protected learning within their work‐base. Clinical placements are a necessary component of the NA programme to enhance general knowledge and clinical skills to enable a versatile supplement to the nursing profession, capable of functioning in many capacities within the health and social care system (NMC 2018a; Robertson et al. 2022). Trainee nursing associates (TNAs) are supported by both practice and academic assessors, who work collaboratively with each TNA to ensure appropriate opportunities are provided to develop their knowledge and clinical skills (NMC 2018b).

The implementation of the NA role in 2016 was received positively by those who had been unable to enter nursing education due to either education or financial limitations (Burnell 2015; King et al. 2022). In the first wave of cohorts, 70% of TNAs explicitly stated their intention was to continue their education and become a registered nurse (Vanson and Bidey 2019; King et al. 2023). However, both personal and organisational challenges have been identified (King et al. 2020). Acceptance of the new role, ambiguity of responsibilities, scope of practice and where the role fits within the nursing structure has caused concern (Green 2019; Lucas et al. 2021; King et al. 2020). There is considerable variation of clinical skills practised by NAs across specialities and settings (Kessler et al. 2021).

Furthermore, the exploration of the experiences of the first cohorts of TNAs identified a lack of a clear occupational identity, a lack of understanding of their trainee status and scope of practice (King et al. 2020). The aim of this study was to explore trainee nursing associates' lived experiences of the NA programme to understand if this role has gained clarity and has become embedded within the nursing workforce across health and social care settings in England.

## 2 Methods

The Consolidated Criteria for Reporting Qualitative Research checklist (COREQ) was adhered to in the development of this paper (Tong et al. 2007).

### 2.1 Study Design

Inductive phenomenology influenced the qualitative study design, with a focus on the work of Heidegger (1962) and Groenewald (2004). Heideggerian philosophy guides the exploration of the essence and meaning of being through a process of understanding an individual's lived experience. This process includes an interpretation of an individual's understanding of their world and a particular phenomenon through language (Heidegger 1962). The Heideggerian approach supported the development of this study to explore TNAs their experiences through using their own language, time to interpretate their experiences, make sense of their feelings and how their lived experience influenced their future expectations and aspirations.

### 2.2 Sample

Recruitment for the study was supported by purposive sampling strategy. Inclusion criteria included learners who were completing an NA programme delivered in a higher education institute in the West Midlands, England, and were interested in being involved in a focus group. Exclusion criteria included learners who had not completed a clinical placement or were within the first 6 months of their programme. Potential participants were asked to contact the last author directly for further information and discussion. Four focus groups were completed, with a total of 14 participants (refer to Table 1). All the names of participants within Table 1 and throughout this manuscript are pseudonyms to protect the confidentiality and anonymity of participants.

**TABLE 1:** Overview of participants.

Focus group	Name^a^	Previous role, length of experience and speciality
17/06/2021	Jane	HCA for over 10 years in emergency care
	Ivy	HCA in care homes and orthopaedics
24/06/2021	Lily	HCA for 7 years supporting people with learning difficulties
	Olivia	HCA for 12 years in care homes and orthopaedics
	Isla	HCA in a doctor's surgery and outpatients
	Henry	Ambulance technician for 6 years and HCA in emergency care
21/10/2021	Ava	HCA for 10 years in mental health, dementia and palliative care
	Emily	HCA for 20 years in district nursing
	Maya	HCA in older people care in hospital
	Launa	HCA for 10 years in surgery
04/11/2021	Sophie	Completely new to healthcare
	Aisha	Completely new to healthcare
	Ruby	HCA for 17 years in sexual health
	Anna	HCA for 15 years in mental health

Abbreviation: HCA, healthcare assistant.

^a^ All names are pseudonyms.

### 2.3 Participants

Of the 14 participants, 13 were female; all participants had completed a year of their programme, and the majority had significant healthcare experience ranging from six to 20 years. Only two participants were new to healthcare and had been employed to complete the NA programme whilst working as a healthcare assistant (HCA).

### 2.4 Data Collection

Data collection occurred through focus groups, which were specifically designed to explore the role of a TNA, their experiences and expectations during their programme. Participants were recruited from four cohorts, where there was the possibility of existing relationships and shared context, which supported a deeper understanding of the topic (Githaiga 2014; Brown 2015). The first two focus groups were conducted online through MS Teams, due to COVID‐19 restrictions, and the last two focus groups were conducted in person on campus.

A semi‐structured interview guide for the focus groups was developed from published literature (Attenborough et al. 2020; King et al. 2020; Roulston and Davies 2019). The first question supported participants to introduce themselves, which was followed by ‘why did you decide to apply to become a NA?’. This was followed by three questions focusing on their role as a TNA, which included ‘can you describe or explain your current role as a TNA?’; ‘how has your role changed since you became a TNA?’; and ‘how do you think your role might develop when you are a NA?’. Finally, the participants experiences and future expectations were explored, which included ‘what were your expectations of this new role before you commenced your training as a NA?’ and ‘how have your expectations of your new role changed during your training to become a NA?’. Each question was supported with follow‐up questions to explore participants' experiences in‐depth. All focus groups were audio recorded and then transcribed verbatim by the first author; focus groups ranged from 31 to 47 min.

### 2.5 Data Analysis

The data were analysed from an inductive phenomenological perspective through the application of thematic analysis as described by Braun and Clarke (2006). The six phases of thematic analysis included the reading and re‐reading of each focus group transcript by the first and last author. Second, the relevant elements of each transcript were coded before moving onto the third stage and the development of codes into themes. On completion of this stage, each theme and assigned codes were re‐explored to ensure representation and the essence of the data; all authors were involved in this process. Finally, the names and a constructive understanding across themes were developed.

### 2.6 Ethical Considerations

Ethical considerations formed the approach of our research; the authors involved in recruitment, facilitation of focus groups and transcription of anonymised transcripts had no previous contact with the participants. Therefore, participants freely volunteered with an understanding that their lecturers would not know who did or did not participate. Authors who deliver the NA programme supported the development of this research and the data analysis of anonymised transcripts.

Ethical approval for this study was obtained from the Health, Education and Life Sciences Faculty Academic Ethics Committee at Birmingham City University on 11 May 2021 (Brooke/#9456/2021/Mar/HELS FAEC). All participants received a participant information sheet, were provided with the opportunity to ask questions prior to the provision of informed consent and reminded participation was voluntary, and if they did or did not participate, this had no impact on their ongoing studies.

### 2.7 Rigour

Rigour was supported by addressing the four elements of credibility, dependability, confirmability and transferability as described by Lincoln and Guba (1985). Credibility and transferability were supported by the inclusion and exploration of trainee nursing associates' experiences, the use of direct quotes within our findings, and data saturation was reached. Dependability was supported by the collection of rich, in‐depth qualitative data by the first author and the engagement of all authors in data analysis and in‐depth discussions to resolve any differences in opinions. Confirmability was supported through the process of adhering to a Heideggerian approach of inductive phenomenology (Heidegger 1962) and the recognition of preconceptions as they arose, which supported the prevention of bias within data analysis.

## 3 Findings

Four themes were identified: new opportunities and knowledge; academic and practice support; pressure within clinical placements; and the need for continued education and training. Each theme is discussed in‐depth below.

### 3.1 New Opportunities and Knowledge

Participants viewed their TNA role as a valuable chance to expand their knowledge through new opportunities and acquire new clinical skills and transition from being an HCA to a TNA. For example, Ivy discussed the development of her role on a surgical ward and the new opportunities to acquire new skills, whilst Henry discussed becoming involved in working with a research team and Jane identified how she had become responsible and accountable for handovers from ambulance crews.

My role is changing, although I do still work as an HCA and I find that I'm doing more, I help with admissions and do all the admission paperwork, and then I have the routine post‐ops and the paperwork side of things and then I have the drugs rounds. Ivy

Well, now I can work with the research team, which was a massive learning curve for me. We also get to work with the major stroke team, and get to walk the pathway with the patient, from coming into hospital and being admitted, including through all the scans and interventions. So that was big learning curve for me. Henry

So, the difference now between being a HCA and a TNA is I'm doing handovers, I'm taking the handover from the ambulance crews, I have become part of the team completely … So, I'm quite happy, it's a big difference, because I have more responsibility now. Jane

The development of participants' role from an HCA to a TNA supported Ivy, Henry, Jane and other participants in beginning to feel as they were an integral part of the multidisciplinary team. However, becoming confident in new clinical skills was identified as a gradual process, and when provided with time to develop their skills, participants welcomed their new responsibilities, as Ivy discussed.

It's very hard to find the time, especially at the beginning when I felt I didn't have the knowledge and the capabilities. I found it very difficult at first, but now that I am more able to kind of go off and do something and then come and get it checked, it's much better…. Ivy

### 3.2 Academic and Practice Support

Participants discussed the need for support from tutors within the university and registered nurses in practice, which was considered essential to successfully progress through the NA programme. Participants identified times when both academic tutors and registered nurses had provided helpful and unhelpful support. For example, Ava identified academic tutors as planned and organised when compared to support within practice, whilst Launa expressed frustration that she did not receive more initial academic support, especially as this impacted on her ability to learn.

… university is on time. It's clearly planned. It's (information) uploaded to support, they reply to their emails, and I think when you get used to that support and then you go into like a real‐life situation, you're not getting it. Ava

We're not supported initially … I'm still waiting for things; they know I don't have the tools to successfully do this course. They tell me to send the e‐mail, and I'm still waiting for the required responses. Launa

Clinical practice support was also discussed both positively and negatively, as Ivy identified working with a qualified nurse on her designated learning days in practice, and even outside of these hours supported the development of her clinical skills. Whilst other participants expressed their dissatisfaction with not being supported, as Ava identified, there was a lack of recognition of her years of experience in healthcare.

I feel like I get quite a lot of support in practice and a lot of encouragement from the nurses. When we're on a learning day, we get partnered with a nurse and they're quite good at supporting us to do new things. But even when I'm not on a learning day, I just have to ask, and the nurses are more than willing to support me. Ivy

I've worked in community care for many years, so I know clinical paperwork … those things come natural to me, you don't have to teach me … I just think it's a lack of understanding of the role, our experience, and our capabilities. Ava

### 3.3 Pressure Within Clinical Placements

A significant concern of participants was the support within and structure of clinical placements. Four overarching concerns were raised by participants; first, a placement on a busy clinical ward, which was short‐staffed, impacted both the development of participants clinical skills and how they supported their patients. Busy clinical placements, which were short‐staffed, led to participants waiting for a registered nurse to provide supervision and patients waiting for care, as Jane identified:

Sometimes when the patient will say ‘oh can you do this’. I say I need to double check because I will not take any decision, if the staff nurse is busy with her own patient, you need to wait and when it's short staffed, you wait a long time. Jane

Second, participants were not always recognised as a learner and a supernumerary learner on clinical placement. A lack of recognition was due to traditional nursing students being provided with more opportunities to learn. The lack of recognition by registered nurses and their approach to favour nursing students impacted on our participants who felt they had become invisible, which led to missed opportunities to learn and develop their clinical skills, as Anna described:

There needs to be more awareness of our role, because what happens in placement is that nurses don't even recognize we are students and when you actually have another normal student (nursing student) with the university logo and everything, they will take and teach that student and I'm thinking I'm also a student here! Anna

Third, participants all expressed concern of being treated as a HCA, which reinforced their beliefs of not being recognised as a learner. Participants were concerned some nurses assumed they were HCAs completing extra training, and the duties of an HCA were seen as more important than further training. The opportunity to engage in learning was only supported if time allowed, as Launa identified:

I feel I am a HCA every shift, that's the problem and it's because we come out of the same funding as HCAs. So, we are HCAs and when the opportunities arise, you become a TNA. But as an HCA on a busy surgical ward, the duties of an HCA are never finished, so in reality it is impossible to become a TNA. Launa

Fourth, the new cohorts of TNAs included learners who had no previous experience of providing healthcare, as they were directly employed by NHS Trusts as a TNA. Participants identified registered nurses were unaware of this new initiative, which caused friction in clinical placements, as Sophie identified:

There are nurses who sometimes don't want to support you, they say you should know this, they judge you because you are in this role, and ask ‘how are you a TNA if you've never worked in the healthcare? If you have never worked in the hospital before’, that's the questions that I get thrown at me sometimes. ‘How are you doing this if you don't know how to care’. Everyone's got to start somewhere we also have to start somewhere…. Sophie

However, participants recognised the value and importance of clinical placements, and they enjoyed the process, as Olivia identified:

I have learned a lot, especially in going to different places and placement, such as renal dialysis. It's been so nice understanding the importance of dialysis. I've been a carer for about 12 years, and started working in a residential nursing home, I am enjoying learning in these different environments. Olivia

### 3.4 The Need for Continued Education and Training

Most participants had not been interested in traditional full‐time study; commencing the NA programme helped participants overcome barriers to engaging in education, which allowed them to move forward in their careers, as Maya identified:

I didn't know what I wanted to do … I didn't want to be a full‐time student, so I thought this (becoming a TNA) was a nice way of doing it and learning while working. Maya

Participants also discussed the need to continue education and training on completion of their NA programme. One example was the need to continue to develop their clinical skills, as identified by Jane:

As a TNA … if you're not been signed off to do IV (intra‐venous medications), you are not seen as helpful. So, you must complete extra training … so for me as TNA to build that confidence and to move on slowly with becoming a team member, this is required. Jane

Many of the participants identified the development of new clinical knowledge and skills as one of the reasons for wanting to continue engaging in education and training. These participants expressed their willingness to continue university studies to become a registered nurse, as identified by Laura:

Ultimately now, the end goal is to be registered nurse. This (NA programme) was the best opportunity for me, to commence my studies, and now I want to continue. Launa

## 4 Discussion

Our study explored TNAs' lived experiences of the NA programme to understand if this new role had begun to be embedded within the nursing workforce across health and social care in England. Four themes were identified: new opportunities and knowledge; academic and practice support; pressure within clinical placements; and the need for continued education and training, which will be discussed with relevance to wider literature.

### 4.1 New Opportunities and Knowledge

The implementation of the NA programme was timely for many participants within our study as previously, there were limited options to support their professional advancement (Wakefield et al. 2009; Snyder et al. 2018; Health Education England [HEE] 2019). The NA programme offered a cost‐effective pathway supporting participants to develop their career and step into a recognised nursing role (Coghill 2018a, 2018b; King et al. 2020; Vanson and Bidey 2019). This was an important element identified by our participants, as they became responsible and accountable for their clinical practice, by adhering to the Nursing Midwifery Council (NMC) standards and becoming a registrant with the NMC (NMC 2018a).

The development and implementation of the NA programme has supported widening participation within HEIs, and the collaboration with health and social care providers has supported both HCAs and the nursing workforce (Boeren and James 2017; Burnell 2015). The provision of the NA programme commenced with the recruitment of HCAs, who were encouraged to apply by their employers, as the apprenticeship model supported employers to gain financial incentives (Education and Skills Funding Agency 2021; King et al. 2023). Due to this process, the NA programme has been identified to improve staff retention by the process of ‘growing your own’ nursing workforce (HEE 2020).

### 4.2 Academic and Practice Support

The implementation of the new role of NA within health and social care originally caused confusion and a lack of familiarity with the role of a NA, requirements of the programme and TNAs as learners. These uncertainties created challenges for employers (Vanson and Bidey 2019; Kessler et al. 2020) and learners (Coghill 2018a, 2018b; Vanson and Bidey 2019; King et al. 2020). Our study identified the continued lack of support and recognition of TNAs as learners in some clinical placements (Vanson and Bidey 2019; King et al. 2020). A major concern, which also continued, was the expectation of a TNA to work as an HCA (Coghill 2018a, 2018b; Vanson and Bidey 2019; King et al. 2020). A new initiative, which may support this concern, is the National Healthcare Uniform that was launched in Autumn 2023.

The role of the NA has begun to be embedded in health and social care settings through the education of healthcare professionals and promotion of the role (Kessler et al. 2021; Robertson et al. 2022). This approach has begun to address any misconceptions or misunderstandings of healthcare professionals. Due to the lack of staff in some clinical placements, participants in our study still believed registered nurses were reluctant to invest their time in supporting them, which was previously reported by King et al. (2020). Registered nurses were also reported to continue to ‘favour’ nursing students and the lack of staff prevented sufficient learning opportunities for TNAs. Unfortunately, the lack of timely and meaningful support in clinical practice remains a real concern for TNAs (Attenborough et al. 2020; King et al. 2020; Lucas et al. 2021; Robertson et al. 2021).

### 4.3 Pressure Within Clinical Placements

The participants within our study identified pressures within clinical placements. TNAs whose work base was in community health and social care identified clinical placements in acute hospital settings as both challenging and daunting, which has previously been identified (Robertson et al. 2023). Furthermore, TNAs identified their further training needs to engage in clinical placements significantly different from their previous scope of practice as often ignored (Robertson et al. 2023). The impact on TNAs was further enhanced due to the focus of education provided in HEIs on secondary care, due to the expectation that the majority of TNAs originated from secondary care settings (Vanson and Bidey 2019; Kessler et al. 2020). An issue that needs to be addressed across nursing programmes.

A further challenge identified by our participants and those of King et al. (2020) was the terminology applied within the NA programme. For example, nursing students were ‘supernumerary’; TNAs were provided with ‘protected learning time’. The use of different terminology was confusing to healthcare managers and registered nurses, across primary and secondary healthcare services, who did not appreciate the time commitment required by TNAs to learn new clinical skills (Kessler et al. 2020; King et al. 2023). However, trust and value earned over time between NAs and registered nurses has begun to address this issue and establish the role of the NA within nursing and healthcare teams (Lucas et al. 2021).

### 4.4 Need for Continued Education and Training

Participants within our study identified the need for continued education and training, which included the development of further clinical skills and responsibilities. The development of further clinical skills varied depending on the speciality of NAs workplace, such as critical care (Bates 2019), aesthetic practice (King 2021) and supporting people with dementia in acute hospitals (Smith and Gill 2020). The different clinical skills acquired by NAs in different specialities have caused some uncertainty regarding the role of the NA, causing both TNAs and registered nurses' confusion and frustration (Kessler et al. 2021). The development of NAs with specific skills within different specialities has also begun to change the generic nature of the NA role.

Many of our participants aim was to become a registered nurse, through the completion of further education, such as the registered nurse degree apprenticeship (NHS 2023). Our participants, however, were uncertain about how and where to apply for such training (King et al. 2020). The development and implementation of the NA programme was to also increasing access to the nursing profession (Coghill et al. 2018a, 2018b; King et al. 2020), which appears to have been successful.

### 4.5 Limitations

The limitations of our study are the inclusion of TNAs from only one HEI and completing their studies during the COVID‐19 pandemic and were mainly female. These limitations were addressed as our participants were employed by NHS and private healthcare providers and had a range of experiences within healthcare prior to commencing their NA programme.

### 4.6 Wider Implications

The complexities of embedding a new role within the nursing workforce have been recognised internationally, although most of the research focuses on the role of nurse practitioners (Aerts et al. 2020; Black et al. 2020; Kerr and Macaskill 2020; Taylor et al. 2021). The implementation of a new role within the nursing workforce requires integrated interprofessional collaboration (Aerts et al. 2020). Our study also identified a need for a clear and consistent description, focus and responsibilities assigned to the new role across settings. The requirement of both relevant policies and structures to support the implementation of the new role has been identified (Black et al. 2020). Our study suggests a cultural acceptance, especially within nursing, is also required; otherwise, there is a risk of undervaluing the new nursing role. The need to support the development of a professional identity of those within the new nursing role has been identified as essential (Kerr and Macaskill 2020) through the development of standards and competencies (Taylor et al. 2021). Our study also identified the need for professional identity through unique uniforms, professional groups, consistent terminology and recognition of knowledge as well as competencies of those undertaking a new role.

## 5 Conclusions

The apprenticeship model of the nursing associate programme has supported many learners to engage in an affordable professional nursing pathway. The nursing associate programme has supported healthcare assistants to progress their career, and many are now focusing on continuing their studies to become registered nurses. However, challenges remain in the identification of meeting the needs of trainee nursing associates in clinical practice, including recognising individual's needs, but also providing equitable supervision for training nursing associates and nursing students. The development of the nursing associate role in different specialities is widening the role of the nursing associate, but simultaneously creating confusion on the responsibilities of nursing associates and will ultimately change the original purpose of the role, which was to be generic across specialities.

## Author Contributions

All members of the team were involved in the development of the paper, S.P. supported the development of the introduction, S.S., K.S. and V.S. worked collaboratively on the development of themes and writing the findings, J.B. was responsible for writing the methods, developing the study, recruiting participants and facilitating focus groups and, finally, J.B. and K.S.‐H. developed the discussion with guidance from the rest of the team. All authors have approved the final submitted version of this paper.

## Conflicts of Interest

One member of the team was directly responsible for supporting the nursing associate programme within higher education. However, this author did not support the recruitment of participants, the facilitation of focus groups or the transcription of data into anonymised manuscripts.

## Data Availability

The data that support the findings of this study are available on request from the corresponding author. The data are not publicly available due to privacy or ethical restrictions.
